# Digital Interventions to Support Adolescents and Young Adults With Cancer: Systematic Review

**DOI:** 10.2196/12071

**Published:** 2019-07-31

**Authors:** Lisa McCann, Kathryn Anne McMillan, Gemma Pugh

**Affiliations:** 1 Digital Health and Wellness Group Department of Computer and Information Sciences University of Strathclyde Glasgow United Kingdom; 2 Centre for Sports and Exercise Medicine William Harvey Research Institute Queen Mary University of London London United Kingdom

**Keywords:** adolescent, neoplasms, telemedicine, systematic review, eHealth

## Abstract

**Background:**

The last decade has seen an increase in the number of digital health interventions designed to support adolescents and young adults (AYAs) with cancer.

**Objective:**

The objective of this review was to identify, characterize, and fully assess the quality, feasibility, and efficacy of existing digital health interventions developed specifically for AYAs, aged between 13 and 39 years, living with or beyond a cancer diagnosis.

**Methods:**

Searches were performed in PubMed, EMBASE, and Web of Science to identify digital health interventions designed specifically for AYA living with or beyond a cancer diagnosis. Data on the characteristics and outcomes of each intervention were synthesized.

**Results:**

A total of 4731 intervention studies were identified through the searches; 38 interventions (43 research papers) met the inclusion criteria. Most (20/38, 53%) were website-based interventions. Most studies focused on symptom management and medication adherence (15, 39%), behavior change (15, 39%), self-care (8, 21%), and emotional health (7, 18%). Most digital health interventions included multiple automated and communicative functions such as enriched information environments, automated follow-up messages, and access to peer support. Where reported (20, 53% of studies), AYAs’ subjective experience of using the digital platform was typically positive. The overall quality of the studies was found to be good (mean Quality Assessment Criteria for Evaluating Primary Research Papers from a Variety of Fields scores >68%). Some studies reported feasibility outcomes (uptake, acceptability, and attrition) but were not sufficiently powered to comment on intervention effects.

**Conclusions:**

Numerous digital interventions have been developed and designed to support young people living with and beyond a diagnosis of cancer. However, many of these interventions have yet to be deployed, implemented, and evaluated at scale.

## Introduction

### Background

Globally, it is estimated that approximately 1 million adolescents and young adults (AYAs) between the ages of 15 and 39 years are diagnosed with cancer each year [1]. Continual advances in cancer therapies now mean that the overall 5-year cancer survival rate among AYAs has increased to more than 80% with survival among some cancer diagnoses (eg, Hodgkin lymphoma, melanoma, and thyroid carcinoma) now exceeding 90% [2]. However, young people who have been diagnosed with cancer often face a myriad of physical, emotional, and psychosocial challenges because of their diagnosis and treatments [3,4]. During treatment, young people often experience prolonged periods of hospitalization and a number of symptoms and side effects such as neutropenia, nausea, alopecia, mucositis, and neuropathy. Post treatment, in survivorship, there is substantial evidence that AYAs diagnosed with cancer are at increased risk of developing long-term health conditions and experience high levels of pain, fatigue, and poor quality of life throughout their life course [5-7]. These difficulties are challenging for AYAs living with and beyond a diagnosis of cancer to manage and are faced at a time when they, as young people, should be establishing independence and autonomy [8,9]. Continual efforts are, therefore, being made in cancer care, research, and policy to ensure AYAs diagnosed with cancer receive the specialist medical, emotional, and practical support they require both during and after their cancer treatment [4,10,11].

Electronic health (eHealth), mobile health (mHealth; interventions delivered using mobile devices), and digital health interventions apply modern computing and technology innovations in the context of health care provision (the encompassing term *digital health interventions* has been adopted for the purpose of this review) and have been proposed as strategies to support young people with cancer manage the challenges associated with their diagnosis and treatment [12-14]. This is significant for AYAs in the context of their *digital native* status; for this population, continued exposure to and integration of digital interventions is the norm [15]. In the context of cancer, digital health interventions have the potential to widen access to and reach of support available to young people with cancer, particularly those being treated as outpatients or receiving long-term follow-up care. Moreover, the delivery of self-directed interventions remotely through digital technology has the potential to ease pressures on face-to-face services and overcome typical geographical and time-related constraints faced by patients, issues particularly pertinent among young people living with and beyond a diagnosis of cancer [16,17].

As demonstrated within the narrative review by Devine et al [18], there now exists a diverse range of digital health interventions for young people with cancer, which contain a variety of elements and functions. This is positive and reflects AYAs’ preferences for information resources and self-management tools relevant to their diagnosis and experiences of cancer to be made available in digital formats [19-21].

In the digital health context, previous reviews of digital interventions have focused on health behavior change and have identified a number of key components that influence intervention outcomes. Existing reviews of digital interventions targeting health behavior change suggest user involvement in intervention design, mode of delivery (eg, Web-based, mobile based, through an advisor, telephone, or e-mail), synergistic use of behavior change techniques, and usability (ie, how easy is the digital health intervention to use and engage with) heavily influence intervention outcomes [22,23]. In this review, assessing these same components and also the quality (ie, the engagement, functionality, aesthetics, and subjective appeal) of interventions is progressive and allows the utility of digital health interventions for AYAs diagnosed with cancer to be more definitively established. Moreover, assessing factors, which influence the engagement and compliance of AYAs living with and beyond a diagnosis of cancer with digital health interventions provide important insights into the feasibility of delivering self-directed interventions to this population in digital formats [24,25]. Understanding which component features of digital health interventions are most acceptable to AYAs diagnosed with cancer and whether such components affect intervention outcomes is critical to the development and evaluation of further digital interventions for young people with cancer [26]. Such data can be used to inform the design, development, and implementation of high-quality effective digital health interventions designed for AYAs diagnosed with cancer.

### Objectives

The objective of this review was to identify, characterize, and fully assess the quality, feasibility, and efficacy of existing digital health interventions developed specifically for AYAs living with and beyond a diagnosis of cancer.

This review aims to address the following questions:

1. What types of digital health and technology intervention have been used to support AYAs diagnosed with cancer? What is their primary focus?
2. Have digital health interventions designed to support AYAs living with and beyond a cancer diagnosis been thoroughly developed and tested?
3. What is the uptake and reach of digital health interventions designed to support AYAs diagnosed with cancer?
4. Is there sufficient evidence to state digital health interventions are an effective means to support AYAs diagnosed with cancer?

## Methods

### Overview

The full protocol for this review has previously been published [27]. To summarize, a literature search for digital health and technology interventions developed specifically for or piloted among AYAs diagnosed with cancer was conducted. Digital health interventions for the purpose of this review encompassed any eHealth, mHealth, or digital health effort, which applied modern computing and communication methods. The review was carried out following the Preferred Reporting Items for Systematic Review and Meta-Analyses guidelines [28].

### Eligibility Criteria

Studies were eligible if they were written in English and published in a peer-reviewed journal and reported or described any existing digital health intervention designed specifically for young people, aged 13 to 39 years, diagnosed with cancer. In this review, digital health interventions include any eHealth, mHealth, or digital health effort, which applied modern computing and technology innovations in the context of health care provision. Participants of interest are those aged between 13 and 39 years, defined as teenagers, adolescents, or young adults living with or beyond a cancer diagnosis, and this was inclusive of survivors of pediatric cancer who fell within the age bracket of interest.

Studies were excluded if they had insufficient detail on the target population or included an incomplete and vague description of the digital health intervention of interest. If a study reported on interventions developed for young people with comorbid conditions other than cancer or if young people with cancer were not the main focus of the study, then the study was excluded. Studies that focused on the use of digital health interventions by parents or survivors of cancer over the age of 40 years were excluded, as were studies where the mean age of the sample was over 39 years.

### Search Strategy

Bibliographic databases (PubMed, Web of Science, and EMBASE) were searched in August 2016 and again in October 2017 for articles written in English and published to date in peer-reviewed scientific journals. A combination of Medical Subject Heading terms and keywords was used. These are available in Multimedia Appendix 1.

### Selection of Studies

GP and LM screened the titles and abstracts of all studies identified during the search using the predetermined eligibility criteria of any study. The interrater agreement between both authors on the eligibility was high (Cohen kappa >0.90), and any instances of disagreement were resolved through discussion.

### Data Extraction

Data extraction was conducted by all authors using a template designed to collate details on each digital health intervention. Data included (1) study characteristics (country, design, sample size, target population, recruitment setting, aim, and methods), (2) platform development and design process (steering committee and patient and public involvement), (3) digital health intervention primary outcomes (mean change and effect size if applicable), and (4) feasibility of delivering the intervention (acceptability, compliance, recruitment response, and retention to the intervention). The mode of digital health intervention delivery was coded into automated functions, communicative functions, and use of supplementary modes based on the coding scheme used by Webb et al [23]. An adapted version of the Mobile App Rating Scale (MARS) was used to group and classify reported engagement, functionality, aesthetics, information quality, and subjective quality of each digital health intervention [29]. Specifically, the theoretical background and strategies scale of the original MARS tool was used to classify the intervention features and theoretical design of each intervention. Alongside data extraction, methodological quality of the included studies was simultaneously assessed using the Quality Assessment Criteria for Evaluating Primary Research Papers from a Variety of Fields (QualSyst) tool [30]. QualSyst includes scoring systems for quantitative and qualitative studies; the maximum summary quality score for qualitative studies is 20 (10 items), and the maximum summary score for quantitative studies is 28 (14 items). Summary quality scores have been reported as percentages of maximum total scores, ranging from 0% to 100%. The higher the percentage score, the better methodological quality of the study, but no studies were excluded based on limited or reduced methodological quality. Following data extraction, included studies were rereviewed by GP, LM, and KM. Any discrepancies were resolved by discussion.

## Results

### Search Results

**Figure 1:**
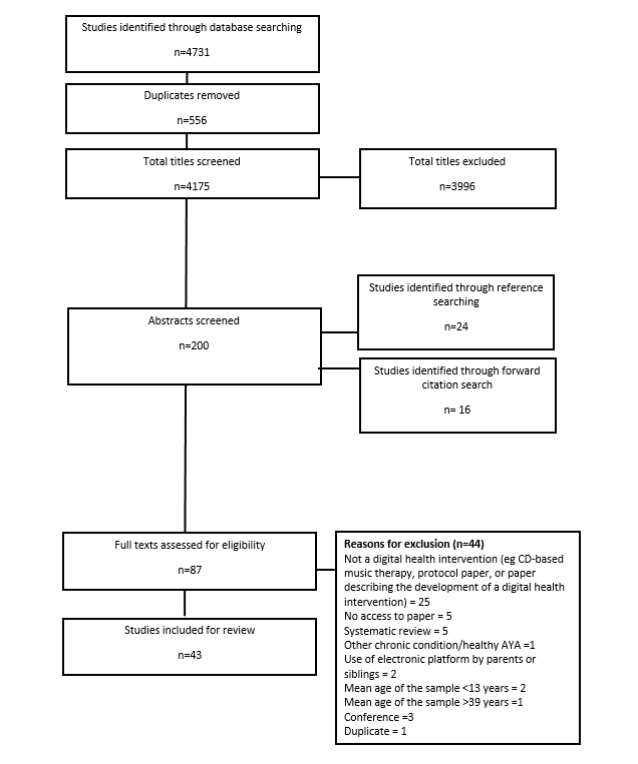
Preferred Reporting Items for Systematic Review and Meta-Analyses flow diagram. AYA: adolescents and young adult.

Figure 1 outlines the search process. A total of 4731 studies were identified through the search. After screening the title of each paper, 195 were identified as potentially eligible, and the abstracts were screened. The full texts of 43 papers describing 38 studies were reviewed and subsequently selected for inclusion.

### Digital Health Interventions Characteristics

The characteristics of each of the 38 studies are summarized in Multimedia Appendix 2. The included 43 papers reporting on these studies were published between 2002 and 2017. A range of study designs was reported. Of the included studies, 12 used a cross-sectional single-group design [31-42], 11 were randomized controlled trials (RCTs) [43-55], 7 were of single-group repeated measures design [56-65], 4 were of qualitative design [66-69], 2 discussed the development of a digital health intervention [70,71], 1 used a mixed-methods approach [72], and 1 was a non-RCT [73]. Sample size ranged from 6 to 375, and age of participants ranged from 10 to 55 years (mean age <33 years). All included study participants were reported within the AYA age range (13-39 years) at the time of diagnosis. The duration of studies ranged from single-use interventions (eg, virtual reality [VR] glasses used during lumbar puncture) [73] to interventions available over long durations (>6 months; eg, Partnership for Health-2, a Web-based smoking cessation intervention, which included a 15-month follow-up) [48].

### Methodological Quality of Reported Studies

Multimedia Appendix 2 outlines the methodological quality of each quantitative and qualitative study. The QualSyst scores are reported as a percentage to allow a comparison to be drawn across study designs, as there are different assessment criteria for quantitative and qualitative studies [30]. Scores ranged from 35% to 100% and were distributed across this range, varying between and within study design. The mean score was 75% for RCTs (n=13) [43-55], 71% for the non-RCT study (n=1) [73], 70% for cross-sectional single-group studies (n=12) [31-42], 74% for repeated measures studies (n=10) [56-65], 62% for platform development studies (n=2) [70,71], 65% for the qualitative studies (n=4) [66-69], and 95% for the studies using a mixed-methods approach (n=1) [72]. Full details of the methodological quality of the papers can be found in Multimedia Appendices 3 and 4.

### Patient and Public Involvement in Design

Of the 38 studies discussed across the 43 research papers included in this review, 14 were designed with young people’s identifiable involvement in the process, and 8 had expert input or included a steering group in the design process.

### Target Behavior

A total of 15 interventions focused on symptom management and medication adherence [33,38,42,52,53,61-63,67,72,73], 8 on self-care [33,35,38,40,44,49,57,58,60], 15 on behavior change [35,37,40,44-47,50,54,64,65,70,71] (of which 6 addressed physical activity behavior [35,45-47,50]), 7 on negative emotions [31,40,44,49,57,58,74], 7 on physical health [33,35,44,46,47,50,63], 5 on anxiety or stress [41,43,49, 50,71,73], 5 on goal setting [35,46,47,54,64], 4 on happiness and well-being [31,40,44,70], 4 on depression [31,40,44,49], 3 focused on relationships [40,66,71], 1 on education [40], and the focus of 1 intervention was to provide entertainment [40]. Across the included studies as a collective, there were multiple occasions where the platform had more than 1 target behavior, as noted throughout this section.

### Intervention Features and Theoretical Design

The features used in the interventions were assessed using the MARS classification tool and are discussed in this section. There were 11 categories including an *other* category. Overall, 5 studies used assessment [42,45,62,63,73], 7 used feedback [32,40,42,45,54,65,66], 16 used information or educational strategies [31-33,37,43-47,51,62-65,68,69], 12 used monitoring or tracking [35,41,44,45,54,61-65,71,72], 8 used goal setting [31,35,40,44-47,54], 9 studies included advice or tips and skills building [44,45,51,56,60,62,65,69,71], and 1 used strengths-based strategies [64]. None of the studies included for review used mindfulness and meditation, relaxation, or gratitude strategies. Furthermore, 14 used strategies characterized as other [31,33,34,38,39,48-50,53,54,57,58,60, 61,70]. Reported theories used to inform the intervention features included the theory of reasoned action [65], the Adolescent Resilience Model [49], the Hope Process Framework [39], the self-regulation model of health and illness [53], social cognitive theory [53], cognitive behavioral therapy [57,58], and the Symptom Management Theory [60].

### Digital Health Interventions for Adolescents and Young Adult Cancer Survivors: Component Features and Outcomes

Multimedia Appendix 5 outlines the mode of delivery used for each digital health intervention, including details on automated and communicative function features within the platform. Table 1 illustrates the different digital health interventions described in the 38 studies (43 papers) included in the review. As shown in Table 1, there were 5 interventions in the other category; these included CD-ROM, computer program, digital storytelling, therapeutic music video, and e-mail. The following section summarizes the key outcome measures and findings from the studies categorized by platform and mode of delivery used. Multimedia Appendix 5 gives more detailed insight into the automated functions, communicative functions, and supplementary modes of communication used in each study.

**Table 1:** Digital health interventions described in the studies which were included in the review (N=38).

Type of digital intervention	Studies, n (%)
Website	20 (53)
Mobile/tablet app	5 (13)
Video game	3 (8)
Wearable	2 (5)
Social media	1 (3)
Virtual reality	2 (5)
Other	5 (13)

#### Websites

There were 20 studies where the digital intervention used was a website [31,36-39,41-45,48,56-58,64,66-69,71]. These studies had a variety of target behaviors and often had multiple outcome measures. Target behaviors included psychosocial and/or quality of life [39,57,58,64], cancer knowledge and symptom management [31,38,43], physical activity and/or physical functioning [44,45,64], fertility [37,71], treatment and medication adherence [48], and co-design and development of a platform [71]. More than half the studies focused on the broader indicators of feasibility evaluations, acceptability, usability, and intervention compliance, but measurement of these indicators varied from study to study with no consistency. Website designs varied from logs and diaries, game-like brain training exercises, written assignments where individual feedback was received from psychologists, weekly tips and tricks, and songwriting and video making exercises [31,36-39,41-45,48,56-58,64,66-69,71].

All studies focusing on physical activity and/or physical functioning saw an improvement [44,45,64]. The one study, using a website platform, that measured feasibility and acceptability of the intervention reported that 86% of participants would recommend the intervention, and 71% were satisfied with the intervention and the information available on the intervention website [45]. The more interactive websites, such as those including writing assignments [31,45], had a positive and stronger effect on psychosocial outcomes and quality of life than the more static interventions, where participants were provided with a treatment summary, contact details of health care professionals, or an electronic journal [43].

Hardy et al [56] reported a wide range of time spent on the website participating in the cognitive training intervention (mean=28.4 min over 12 weeks) with a mean of 11.4 training hours during the 12-week period. Seitz et al [57,58] reported that more than 80% of participants were satisfied with the psychotherapy intervention, and more than 80% indicated that the intervention, involving 10 written assignments, was relatively helpful in relieving the symptoms of posttraumatic stress disorder, anxiety, and depression. Furthermore, 90% of participants said they would recommend the website to a friend [57,58]. Moreover, 1 study described the development of 2 Web-based interventions using co-design [71]. A collaborative Patient Research Partner (PRP) approach was used to develop an internet portal focusing on fertility and sexuality and a self-help Web portal for young people with cancer. The PRPs provided feedback on content, system, and service quality. This led to the adaptation of the program, where the acceptability, feasibility, and functionality of the programs were examined [71]. In this case, users of both programs considered the content relevant and informative, and many expressed satisfaction with the website.

#### Mobile or Tablet App

A total of 5 studies reported using a mobile phone or tablet app [32,33,40,59-63,67,72]. Of the 5 studies, 3 focused on symptom assessment and/or symptom management [32,59-61,72], whereas the other 2 studies focused more specifically on pain [33,62,63,67]. Apps developed to aid symptom assessment and symptom management tended to be positively reported [32,59-61,72], but definitive comparisons are difficult because of the different outcome measures used across these studies. For example, the Memorial Symptom Assessment Scale 10-18 was used in the evaluation of the app used by Rodgers et al [59,60]. Results from this evaluation demonstrated the prevalence of symptoms decreased over time (*P*=.006), but there was no statistical difference over time in relation to symptom distress (*P*=.22) [60]. In another study [32,72], participants completed the investigator-created Computerized Symptom Capture Tool on an iPad to report symptom experiences after their first cycle of chemotherapy. Although acceptability data were not reported [72], it was noted that the app did identify a range of unique symptom clusters in these young adults. Most common symptom clusters were nausea, eating problems, and appetite problems, and the most frequently named priority symptom was nausea [32,72]. In their evaluation of a mobile electronic diary, called mOST, for AYAs with cancer to report daily symptoms of pain, nausea, vomiting, fatigue, and sleep, Baggot et al [61] reported an adherence rate of 97% over the 21 daily symptom reporting period. Encouragingly, high adherence rates were maintained throughout the evaluation period [61].

Mobile apps developed to assess and manage pain were also reported positively in 2 included studies. Pain Squad app by Stinson et al [33] was reported as being easy to use by most users (70.2%) and rated as quick to complete (91.7%). Evaluation of the second generation of this app, PainSquad+, by Jibb et al [62] also reported positive results with good initial adherence of their app at 68.8±38.1%. Some decrease in adherence over time was noted by week 4 though at 39.1±38.1%.

#### Video Games

Overall, 3 studies reported using a video game as the platform for delivering the intervention [50,52,53,56]. The target behavior for each study differed: 1 focused on physical activity or physical functioning [50], another focused on cancer knowledge and treatment adherence [52,53], and another focused on memory, attention, and behavioral function [56].

The use of a video game to address these behaviors was reported as successful across all 3 studies respectively [50,52,53,56]. Cancer knowledge and treatment adherence improved in the Re-Mission video game intervention group [52,53], as they used the game as an educational tool, compared with the control group. Slight improvements in both physical activity and physical functioning measures over a 70-day intervention period were also noted for the intervention group [50]. In addition, the use of a game involving brain training exercises, such as Captain’s Log, found improvements in working memory and attention problems [56]. This study by Hardy et al [56] was the only study using a video game where the feasibility and acceptability of the intervention were assessed. Hardy et al [56] reported compliance data, indicating that young people participated in a mean of 28.4 sessions and 11.4 training hours throughout the 12-week program.

#### Wearables

Wearable physical activity trackers were used in 2 studies, and both studies had a main focus of improving physical activity of participants [35,54]. Of the 2 studies, 1 study simply used a consumer market device, a FitBit, to measure steps and encourage increased activity through monitoring [54], whereas the other study also used FitBits but supplemented this with a study-created private Facebook group that participants could use over the 10-week intervention period [54]. Both studies reported increases in physical activity following the intervention, and 1 reported on the intervention feasibility [54]. This was measured through FitBit wear time (71.5% of the available time) and participant engagement with the Facebook group, where 89.7% of participants joined the Facebook group, 92.3% of those saw at least one post, and 65.4% of those who joined commented on at least one post [54].

#### Social Media

One study used the social media platform Facebook to deliver its intervention [46,47] where the focus was to increase physical activity in participants through educational posts with a focus on behavioral strategies for increasing activity [46,47]. Participants within this study also had access to a separate website with a goal setting and physical activity monitoring (diary) tool. Following the intervention, there was an increase in physical activity of 67 min/week in the intervention group and a significant loss in weight (−2.1 kg, *P*=.004).

#### Virtual Reality

Two studies used VR glasses as the platform to deliver their interventions [70,73]. Of these 2 studies, 1 focused on pain during a lumbar puncture and evaluation of the intervention [73], whereas the other focused on the development of a VR counseling system [70]. Although not significant, pain scores were lower in the VR group compared with the control group, and 77% of users noted that the VR glasses and headphones helped to distract them during the lumbar puncture [73]. Because of poor recruitment, authors in the other study were unable to test and evaluate the VR counseling system they designed [70].

### Other Intervention Types

As shown in Table 1, there were 5 interventions in the other category: CD-ROM, computer program, digital storytelling, therapeutic music video, and e-mail [34,49,51,55,66,64]. The focus of these studies included building resilience [49], symptom management [49,55], cognitive function [34], education [51], social therapy [66], and health-promoting behaviors [64]. All studies were concluded feasible and acceptable to young people with cancer, with the majority reporting good uptake and engagement from participants.

### Young Peoples’ Subjective Experience of Using Digital Health Interventions

A total of 20 studies reported young people’s subjective experience of using the digital health intervention [38,39,43,45,48,50-52,54-58,61-63,66,67,69,72]. Subjective experience was typically measured as user satisfaction or appeal of the contents of the intervention. Within 11 studies, participants reported that they would either use the intervention again or would recommend it to a friend [38,39,48,51,52,55,57,58,61,62,65,67]. Very few studies reported participant’s feedback on areas for improvement or recommendations for further platform developments. Of the studies that reported feedback, it was generally that the platform had technical problems, the visual design was too simple, or that the digital platform for communicating with other young people with cancer did not replace personal connection [66]. There was no clear pattern between intervention characteristics (delivery mode and focus of functional components) and engagement or adherence. Reasons for poor engagement or noncompliance were typically either not reported or attributed to recurrent illness [63,50]. Within 1 website-based study [69], incentives were introduced to improve compliance. Some studies reported differences between the engagement and use of different features: for example, Rabin et al [45] reported that participants viewed pages on physical activity logging pages more often than physical activity tip pages of the intervention website (11.38 days vs 0.5 days). Similarly, Mendoza et al reported differences between participants’ frequency of viewing, commenting, and liking Facebook posts within their intervention (92.3% vs 65.4% vs 50%, respectively).

### Effect Sizes

Only 7 of the 43 articles reviewed provided effect size within the original manuscript. Because of the heterogeneity in outcomes and differences in the characteristics of the intervention, it is not possible to make comparisons between the studies. A table summarizing the relevant data is available on request.

### Reach

The studies included in this review did not specifically report intervention uptake and reach. The total sample size of all studies gives some indication as to the number of people the interventions reached as a whole and the breakdown of where studies were conducted provides some guidance as to the characteristics of those participants. There was a total sample size of 1935 participants across the 38 studies. The majority (n=23) of the digital health interventions were designed in the United States [31,32,34,35,39-47,49,51,54,56,59-61,64-66, 68,73], 3 were designed in Canada [33,62,63,67], 2 in the Netherlands [36,38], 2 in Sweden [37,71], 2 reported multiple sites across different countries [48,52,53], and the country was not reported for 6 studies [50,55,57,58,69,70,72,75].

## Discussion

### Principal Findings

In this review, we have focused our attention on digital health interventions for AYAs diagnosed with cancer. We are not alone in our interest in considering digital health–driven interventions for AYA populations at this specific illness foci level [18] or indeed other relevant areas such as mental health [19], complex health care needs [75], and lifestyle behavior interventions for survivors of child and young adulthood cancer [13]. A recent narrative review of digital health interventions targeting AYA cancer survivors demonstrated the range of digital modalities used to support young people with cancer [18]. Our review has moved beyond the review of Devine et al [18] by not only identifying specific interventions but also drawing out components that contribute to appropriate digital interventions for our target population (AYA diagnosed with cancer). Our use of the Mode of Delivery [23] and MARS criterion [29], respectively, has allowed our synthesis to identify key components that may influence successful uptake of digital interventions to support this population in the future.

As stated in the Introduction section, we posed 4 key questions in this review. We have revisited these questions to frame our discussion.

#### What Types of Digital Health and Technology Intervention Have Been Used to Support Adolescents and Young Adults Diagnosed With Cancer? What Is Their Primary Focus?

We considered the mode of delivery (how the intervention was delivered to recipients) in the included studies and identified that websites were the most often used technology. Website designs and functionalities varied across this most prominent mode of delivery from simple logs and diaries to more interactive communications. Of note, given the review’s inclusion timeline of 1970 to 2017 and the associated developments in the digital landscape in this time, it was observed that only 5 studies used mobile phone or tablet apps, and just 2 studies used wearable technologies as the mode of delivery for their associated interventions. We know that digital health interventions are rising in prominence and are helping to expand, assist, and enhance human activities within the context of health care [76]; therefore, whether this balance shifts in the future as even newer digital health innovations are developed remains to be seen.

The growth of the digital environment and associated digital health technologies are known as *disruptive innovations* [77] because they can lead to diverse, but improved, health outcomes [74]. The focus in some of the included studies in this review on improved health outcomes may explain why the observed target behaviors of the included digital interventions predominately focused on measurable outcomes related to symptom management and medication adherence, self-care, behavior change, and reducing negative emotions.

#### Have Digital Health Interventions Designed to Support Adolescents and Young Adults Living With and Beyond a Cancer Diagnosis Been Thoroughly Developed and Tested?

Studies included in this review were a mixture of randomized controlled trials, small-scale pilot studies, or qualitative explorations, which considered the feasibility and efficacy of digital health interventions developed specifically for AYAs living with or beyond a cancer diagnosis.

Our review illustrated that a range of digital health interventions has been developed for AYAs diagnosed with cancer, but few have actually progressed beyond small-scale piloting. This scalability restriction includes the website-based interventions, which may actually have the potential for wider dissemination than interventions that are hardware dependent for deployment. Moreover, even fewer appear ready for wide-scale implementation in routine care provision to help meet AYAs holistic and supportive care needs.

We did not extract information explicitly relating to any cost-effectiveness evaluations of the included interventions, but we did note during our synthesis that it was rare for a context such as this to feature prominently within any of the included articles. Similarly, it was challenging at times to identify explicit examples of interventions being scaled up and embedded within routine supportive care practices for AYAs with cancer.

Other reviews of digital health technologies have noted that engagement of end users in co-design activities throughout the innovation and development pathway for digital health technologies is variable but essential to ensure long-term use and engagement with developed products [75]. In this review, we noted the involvement of young people in the design and development of the digital health intervention in less than half the studies reviewed. Although less than half of the studies reported on AYAs’ subjective experiences of using the intervention, those that did, reported positive experiences.

#### What Is the Uptake and Reach of Digital Health Interventions Designed to Support AYAs Diagnosed With Cancer?

AYAs are typically referred to as digital natives: their continued exposure to and integration in a digital and electronic world is the norm [15]. Digital health care resources are increasingly desirable, and it is a commonplace for digital natives to be responsive to the use of digital technologies to manage their health care needs [21,78]. We found evidence to further support this position in our review for reasons that are threefold. First, we noted a total recruited sample size of 1935 AYAs across the 38 different interventions assessed within this review. Collectively, this provides a strong indication that there is positive traction for the uptake and reach of digital health interventions for AYAs diagnosed with cancer. Second, acceptability ratings of the digital interventions were reported in 58% of the included papers and were generally high. Finally, compliance rates, as reported in 61% of included papers, tended to be good and often sustained.

We observed across the 38 included studies that 18 interventions were primarily focused on supporting AYAs during active cancer treatment, and 20 were designed more explicitly for use across the long-term survivorship period. This further supports the notion that there is a role for digital health interventions to support AYAs with cancer at all stages of their cancer experience. Previous surveys with AYAs with cancer have identified preferences for digital tools to support experiences from diagnosis onward, including treatment and survivorship [21]. Other work has also highlighted the desire of young adult survivors of cancer of the introduction of digital tools to support self-management behaviors [16].

We are cognizant, however, of the context in which much of this work has been conducted. We noted that the majority of the evidence in this review has been drawn from work originating in the United States (61% of the included papers); therefore, there may be some bias in terms of uptake and reach in this regard.

The expanse now of what may be considered a digital health intervention meant the inclusion criteria in this review was purposely broad to capture a range of digital health interventions designed specifically for AYAs with cancer.

#### Is There Sufficient Evidence to State Digital Health Interventions Are an Effective Means to Support Adolescents and Young Adults Diagnosed With Cancer?

This systematic review highlighted that although there is a large quantity of good quality evidence in the field, drawing conclusive statements about the use of technology is difficult, given the heterogeneity of studies conducted. Our decision to appraise the quality of included studies proved useful, as the overall quality of included studies was found to be good as they had a mean QualSyst score of greater than 68%. Some studies were methodologically noncomparable (eg, qualitative acceptability studies vs RCT trials), but generally, included studies were of good quality. Because of the heterogeneity between studies and descriptive reporting included within most, it was not possible to make conclusive statements about which delivery mode or intervention feature has the largest impact on outcome, engagement, or adherence.

We must be mindful of the different health care models and service provision contexts across the countries in which the studies were conducted (United States, Canada, the Netherlands, Sweden, and the United Kingdom), and associated evidence generated. The variability of these health care models (public, private, and insurance-based models of health care) should be considered too when interpreting the findings from this review. Consideration must also be given to the ethical and clinical challenges of using digital technology within AYA cancer services, as overarching principles of care and obligations to safeguard do not change [79]. This is particularly pertinent in instances where physical or psychosocial risks are captured or identified within the digital intervention, and there is a need for intervention or additional support to be provided to the patient [80]. Similarly, the extent to which digital resources are age-appropriate and tailored to the health literacy needs of AYA cancer patients should be addressed.

In addition, although our review has demonstrated digital interventions do provide opportunities to support AYAs diagnosed with cancer, it is challenging at this stage to definitively state which specific platform health care professionals should adopt or recommend to AYAs they care for. Many interventions have yet to be deployed and implemented at scale. If this status quo remains, care provision will not evolve in tandem with technological developments and the growing digital health landscape in which global health services are increasingly situated. This is a challenge to be addressed by colleagues and peers working across both clinical and research AYA cancer fields. Efforts should focus on international collaborations to drive forward interventions on the cusp of upscaling and capable of providing gold standard evidence. Given the relatively small number of AYA cancer survivors globally, efforts to replicate studies using the same outcome measures in other countries should be made. Testing the impact and effect of digital health interventions for AYA cancer survivors beyond traditional RCT models is increasingly necessary to reflect the pace at which developments are occurring and the agile nature of digital technology. Innovation in the context of methodologies alongside innovation in the context of interventions (point of diagnosis, during treatment, and posttreatment) is going to be essential to best inform digital health implementation within routine care provision in the future.

### Strengths and Limitations

Our review has a number of strengths and limitations. We focused our review attentions on digital health interventions designed specifically for AYAs with cancer, and we used broad age inclusion criteria in this regard, from 13 to 39 years. Although this may seem too broad to some, to ensure our review was inclusive as possible and of international significance and relevance, we drew on a range of relevant cancer policy context definitions of AYA [81]. There were some challenges encountered with this, particularly in terms of papers, which included the older spectrum of our target participants (>26 years). Given the international variations in definitions of AYAs with cancer, papers that included these upper ranges of AYA had to be excluded from the review, as it was not possible for us to readily identify data specifically focused on the population up to 39 years, particularly if the intervention had been developed in the context of a wider adult cancer population. Although we searched a range of databases, these were limited to the most common, and we limited our searches to peer-reviewed articles, thereby excluding gray literature. Also, as we limited our searches to papers published in English language only, this may be considered a limitation by some.

To meet our review objective of identifying, characterizing, and fully assessing the quality, feasibility, and efficacy of existing digital health interventions developed specifically for AYAs living with or beyond a cancer diagnosis, our data extraction process was long and detailed. In addition to our study characteristic extractions, we also used 2 specific rating tools relevant for a focus on digital interventions: Mode of Delivery [23] and MARS [29]. Overall, the Mode of Delivery proved a useful and straightforward tool to use, but we encountered some difficulties with the MARS tool. It became increasingly apparent that for the impact of this tool to be realized, one requires full and ready access to the particular app being reviewed and rated. As we were reviewing the published evidence of digital interventions (and not just digital interventions that were publicly and commercially available on app stores such as others who have used the MARS tool in previous reviews [82,83]), we had only narrative descriptions of the apps or interventions to go by in the papers and on occasion, supplemented with screenshots of aspects of the intervention. We were therefore limited to only reporting selected, but still useful, thematic information. Additional items of the MARS were answered if published detail allowed. Although we persevered with this extraction tool throughout our review process, we were unable to draw insightful conclusions because of omission of detail on user interactivity with the digital health intervention, functional performance, ease of use, and graphic design features within manuscripts. We reflect on this as a limitation of the MARS tool itself as much as our review.

### Conclusions

This review is positive in that it has highlighted that multiple digital health interventions do now exist to support AYAs diagnosed with cancer. The everyday technology-driven environment in which we now live has expedited the development pathway for digital interventions in health care contexts. However, within our review, it was rare to identify innovations that are ready to be or have already been deployed and implemented at scale. We find ourselves with a case of digital health *pilotitis* and efforts now need to shift; therefore, the most robust evidence-based innovations are routinely implemented in clinical practice. There is insufficient evidence to state conclusively which form of digital intervention is the best approach to support young people with cancer. Currently, it is challenging to provide clinicians working directly with AYAs with cancer with definitive options for valid, reliable, and robustly evaluated digital tools and interventions to use as part of their health care services. Therefore, to really establish the impact of digital interventions on health-related outcomes of AYAs with cancer and the economic value of implementing digital interventions on service design and service delivery, future endeavors should prioritize upscaled and robust outcome-driven interventions and associated evaluations.

## Appendix

Multimedia Appendix 1 Medical subject heading terms.

Multimedia Appendix 2 A summary of research studies included in the review.

Multimedia Appendix 3 QualSyst Scores for Qualitative Papers.

Multimedia Appendix 4 QualSyst Scores for Quantitative Papers.

Multimedia Appendix 5 Mode of delivery.

## Abbreviations

AYAs: adolescents and young adults
eHealth: electronic health
MARS: Mobile App Rating Scale
mHealth: mobile health
PRP: Patient Research Partner
QualSyst: Quality Assessment Criteria for Evaluating Primary Research Papers from a Variety of Field
RCT: randomized controlled trial
