# Supplementary material for: Digital Interventions to Support Adolescents and Young Adults With Cancer: Systematic Review
Source: JMIR Cancer. 2019 Jul 31;5(2):e12071. doi: 10.2196/12071 (PMC6693302; doi:10.2196/12071)
Supplement: Multimedia Appendix 1 [file cancer_v5i2e12071_app1.pdf]

MeSH Terms

	Search Algorithm
1	Teen* OR Adolesc* OR Young Adult OR Child
2	Cancer OR Cancer Survivor
3	1 AND 2
4	app OR apps OR application or mobile OR Android OR droid OR iphone OR ios OR blackberry or web OR internet OR portal OR portlet OR microsite OR website OR “web site” OR url OR mhealth OR ehealth OR internet OR online OR digital OR email OR social network OR electronic communication OR e-health OR e-learning OR elearning OR social network OR facebook OR myspace OR virtual world OR short messaging service OR virtual clinic OR computer assisted therapy OR information technology OR electronic communication OR digital divide OR e-mail OR email OR telehealth.
5	3 AND 4
