# Supplementary material for: Digital Interventions to Support Adolescents and Young Adults With Cancer: Systematic Review
Source: JMIR Cancer. 2019 Jul 31;5(2):e12071. doi: 10.2196/12071 (PMC6693302; doi:10.2196/12071)
Supplement: Multimedia Appendix 2 [file cancer_v5i2e12071_app2.pdf]

Multimedia Appendix 2

A Summary of Research Studies Included in the Review

Author (Year)<br><br>Country<br><br>Intervention Name	Sample Size	Age Range (years)<br><br>Mean/ Median Age (years)	Key Target Behaviour	Intervention Platform	Outcome Measure(s)	QualSyst Score (%)
**Randomised Controlled Trials**						
Sabel et al., 2016<br><br>Not Reported<br><br>Not Reported	N =13	7-17<br><br>Mean age: 12.5±2.9	Physical Activity	Nintendo Wii	Physical Activity Levels<br><br>Physical Functioning	96
Mendoza et al., 2017<br><br>USA<br><br>Not Reported	N =59	14-18<br><br>Mean age: 16.6±1.5	Physical Activity	Fitbit Flex<br><br>Facebook Support Group	Feasibility<br><br>Physical Activity<br><br>Quality of Life<br><br>Motivation<br><br>Facebook Engagement	62
Kunin-Batson et al., 2016<br><br>USA<br><br>Not Reported	N =52	15-28<br><br>Mean age: 21.3±3.9	Cancer Knowledge and Understanding	Website<br><br>e-Journal<br><br>Messaging System	Cancer Knowledge<br><br>Anxiety<br><br>Multidimensional Health Locus of Control	65
Jones et al., 2010<br><br>USA<br><br>Conquering Cancer Network	N =65<br><br>I =35<br>C =30	12-18<br><br>Mean age: 14.8±1.96	Education	CD-ROM	Frequency and Duration of Use<br><br>Multidimensional Health Locus of Control<br><br>Quality of Life<br><br>Self-Efficacy<br><br>Avoidance<br><br>Coping Strategies<br><br>Approach Help<br><br>Cancer Knowledge	69

Multimedia Appendix 2

**Author (Year)**<br><br>**Country**<br><br>**Intervention Name**	**Sample Size**	**Age Range (years)**<br><br>**Mean/ Median Age (years)**	**Key Target Behaviour**	**Intervention Platform**	**Outcome Measure(s)**	**QualSyst Score (%)**
Akard et al., 2015<br><br>Not Reported<br><br>Not Reported	N =28	7-17<br><br>Mean age:<br><br>Parents =36<br><br>Children =11	Symptom Management<br><br>Quality of Life	Digital Story Telling	PedsQL V4.0	81
Beale et al., 2007; Kato et al., 2008<br><br>USA, Canada, Australia<br><br>Re-Mission	N =375<br><br>I =197<br>C =178	13-29<br><br>Mean age:<br><br>I =15.79<br>C =16.06	Cancer Knowledge<br><br>Adherence to Treatment	Video Game (Re-Mission)	Treatment Adherence<br><br>Self-Efficacy<br><br>Cancer Knowledge<br><br>Perceived Stress<br><br>Health Locus Control<br><br>Quality of Life<br><br>FACT-G	81, 100
Burns et al., 2009<br><br>USA<br><br>SMART Study	N =12	11-24<br><br>Mean age: 17.5	Building Resilience	Therapeutic Music Video	Feasibility<br><br>Symptom Experience<br><br>Symptom Related Risk<br><br>Individual Risk<br><br>Individual Protective<br><br>Resilience<br><br>Quality of Life	73
Emmons et al., 2013<br><br>USA, Canada	N =374<br><br>I =132<br>C =242	18-55<br><br>Mean age: 32±7.9	Smoking Cessation	Website	Smoking Cessation<br><br>Quit Attempts	92

Multimedia Appendix 2

Author (Year)<br><br>Country<br><br>Intervention Name	Sample Size	Age Range (years)<br><br>Mean/ Median Age (years)	Key Target Behaviour	Intervention Platform	Outcome Measure(s)	QualSyst Score (%)
PFH-2					Readiness to Change	
Huang et al., 2014<br><br>USA<br><br>Fit4Life	N =38	10-16<br><br>Median age: 13	Obesity Treatment	Website<br><br>SMS	Weight<br><br>BMI-Z<br><br>Physical Activity<br><br>Diet<br><br>Psychological Behaviours	79
Rabin et al., 2011<br><br>USA<br><br>Not Reported	N =18	18-39<br><br>Mean age: 32.17	Physical Activity	Website	Feasibility and Acceptability<br><br>Physical Activity<br><br>POMS<br><br>Fatigue	46
Valle et al., 2013; Valle et al., 2015<br><br>USA<br><br>Not Reported	N =86	21-29<br><br>Mean age: 31.7	Physical Activity	Facebook	Physical Activity<br><br>BMI<br><br>FACT-G	68,58
**Non-Randomised Controlled Trials**						
Wint et al., 2002<br><br>USA<br><br>VR Glasses	N =30<br><br>I =17<br>C =13	10-19<br><br>Mean age: 13.6	Pain	VR Glasses	Pain<br><br>VR Evaluation	71
**Single-Group Repeated Measures**						
Seitz et al., 2014a; Seitz et al., 2014b<br><br>Not Reported<br><br>Onco-STEP	N =20	20-36<br><br>Mean age: 27.3±4.8	PTSD	Website	Intervention Satisfaction<br><br>PTSF Symptoms<br><br>Anxiety<br><br>Depression	85, 85

Multimedia Appendix 2

**Author (Year)**<br><br>**Country**<br><br>**Intervention Name**	**Sample Size**	**Age Range (years)**<br><br>**Mean/ Median Age (years)**	**Key Target Behaviour**	**Intervention Platform**	**Outcome Measure(s)**	**QualSyst Score (%)**
					Fear	
Hardy et al., 2011<br><br>USA<br><br>Captain’s Log	N =9	10-17<br><br>Mean age: 13.3±2.4	Memory<br><br>Attention<br><br>Behavioural Function	Game	Feasibility<br><br>Working Memory Index<br><br>Digit Span<br><br>Letter Numbering Sequencing<br><br>Inattention Index	50
Gilliam et al., 2011<br><br>USA<br><br>Not Reported	N =12	6-18<br><br>Mean age: 12.75	Physical Function<br><br>Quality of Life	Website	Adherence<br><br>Endurance<br><br>Strength<br><br>Functional Mobility<br><br>Quality of Life	73
Berg et al., 2014<br><br>USA<br><br>Not Reported	N =24	18-34<br><br>Mean age: 23.38	Health Promotion Behaviours	e-Mail	Intervention Acceptability and Feasibility	77
Baggott et al., 2012<br><br>USA<br><br>mOST	N =11	13-21<br><br>Mean age: 18.2±2.9	Symptom Management	App	Feasibility<br><br>Adherence	94
Rodgers et al., 2013; Rodgers et al., 2014<br><br>USA<br><br>EAT!	N =16	11-18	Symptom Management	App	Memorial Symptom Assessment Scale<br><br>Albumin<br><br>Prealbumin	43

Multimedia Appendix 2

Author (Year)<br><br>Country<br><br>Intervention Name	Sample Size	Age Range (years)<br><br>Mean/ Median Age (years)	Key Target Behaviour	Intervention Platform	Outcome Measure(s)	QualSyst Score (%)
					Acceptability and Usability<br><br>Competency	
Jibb et al., 2017; Stinson et al., 2013<br><br>Canada<br><br>Pain Squad+	N =40	12-18<br><br>Mean age: 14.2±1.7	Pain	App	Feasibility<br><br>Pain	82, 80
**Cross-Sectional, Single Group Studies**						
Hooke et al., 2016<br><br>USA<br><br>FitBit	N =17	8-15<br><br>Mean age: 8.69	Physical Activity<br><br>Fatigue	FitBitR	Validity and Reliability<br><br>Responsiveness	68
Stinsen et al., 2015b<br><br>Canada<br><br>PainSquad	Study 1 N =92<br><br>Study 2 N =14	8-18<br><br>Mean age:<br><br>Study 1 13.1±2.9<br><br>Study 2 14.8±2.8	Pain	App	Acceptability<br><br>Compliance	100
Lai et al., 2015<br><br>USA<br><br>SyMon-SAYS	N =57	7-21<br><br>Mean age: 11.9	Fatigue	Website	Feasibility<br><br>Acceptability	80
Kniijnenburg et al., 2013<br><br>Netherlands<br><br>DCOG	N =98	17-55<br><br>Mean age: 31	Medical Decision Making	Website	Usability<br><br>Website Content	60
Kesler et al., 2011<br><br>USA	N =25	7-19<br><br>Mean age: 12.6	Cognitive Function	Computer Program	Compliance<br><br>Cognitive Function	77

Multimedia Appendix 2

**Author (Year)**<br><br>**Country**<br><br>**Intervention Name**	**Sample Size**	**Age Range (years)**<br><br>**Mean/ Median Age (years)**	**Key Target Behaviour**	**Intervention Platform**	**Outcome Measure(s)**	**QualSyst Score (%)**
No Reported						
Wiklander et al., 2017<br><br>Sweden<br><br>Fex-Can	N =23	18-43<br><br>Mean age: 30	Sexual Problems<br><br>Fertility	Website	Demand<br><br>Acceptability<br><br>Preliminary Efficacy<br><br>Functionality	80
Blaauwbroek et al., 2012<br><br>Netherlands<br><br>SCP	N =73	19-56<br><br>Mean age: 38	Information Provision	Website	Website Evaluation	64
Cantrell and Conte, 2008<br><br>USA<br><br>HIP	N =6	21-39<br><br>Mean age: Not Reported	Psychosocial Function	Website	Intervention Evaluation	35
Macpherson et al., 2014<br><br>USA<br><br>C-SCAT	N =72	13-29<br><br>Median age:<br><br>Adolescent 15<br><br>Adults 22	Symptom Management	App	Feasibility and Acceptability	70
McLaughlin et al., 2011<br><br>USA<br><br>LIFE Community	N =14	18-29	Social Support	App	Social Support<br><br>Bridging Social Capitol	85
Suzuki and Beale, 2006<br><br>USA	N =21	13-22<br><br>Mean age: 19.3±2.4	Purpose of Website use	Website	Cancer-Related Information Dissemination	50

Multimedia Appendix 2

**Author (Year)**<br><br>**Country**<br><br>**Intervention Name**	**Sample Size**	**Age Range (years)**<br><br>**Mean/ Median Age (years)**	**Key Target Behaviour**	**Intervention Platform**	**Outcome Measure(s)**	**QualSyst Score (%)**
					Interpersonal Connection	
Wu et al., 2011<br><br>USA<br><br>ESRA-CAF	N =40	13-20	Monitoring Symptoms	Website	Completion Rates	73
**Platform Development**						
Phelps et al., 2016<br><br>Not Reported<br><br>C:EVOLVE	N =12	15-23	VR Counselling System Development	VR	System Development<br><br>System Pilot	55
Winterling et al., 2017<br><br>Sweden<br><br>Fex-Can	N =13	20-41	Website Development	Website	Co-Design Success	60
**Qualitative Studies**						
Donovan et al., 2014<br><br>USA<br><br>Not Reported	N = Not Reported	15-39	Social Support	Website	Types of Social Support	70
Gonzalez-Morkos et al., 2014<br><br>USA<br><br>TI Webcast	N =6	15-18	Social Therapy	Live Webcasting	User Experience and Satisfaction	70
Griffiths et al., 2015<br><br>Not Reported<br><br>Realshare	N =12	16-30<br><br>Mean 21.08±4.15	Website Development and Usage	Website	Experience of Using Realshare<br><br>Acceptability	45

Multimedia Appendix 2

**Author (Year)**<br><br>**Country**<br><br>**Intervention Name**	**Sample Size**	**Age Range (years)**<br><br>**Mean/ Median Age (years)**	**Key Target Behaviour**	**Intervention Platform**	**Outcome Measure(s)**	**QualSyst Score (%)**
Stinson et al., 2015a<br><br>Canada<br><br>Teens Taking Charge: Managing Cancer Online	N =22	12-18<br><br>Mean age: 15.2±1.8	Self-management	Website	User Satisfaction<br><br>Design<br><br>Content<br><br>Functionality and Features<br><br>Sociability<br><br>Desire	75
**Mixed-Methods Studies**						
Ameringer et al., 2015<br><br>Not Reported<br><br>mOST	N =72	13-29<br><br>Mean age: 18.5	Symptom Management	App	Symptoms<br><br>Symptom Clusters	95

Note. SD = Standard Deviation, I = Intervention Group, C = Control Group, BMI – Body Mass Index, VR = Virtual Reality, CD-ROM = Compact Disc Read-Only Memory, PTSD = Post-Traumatic Stress Disorder
