# Supplementary material for: Digital Interventions to Support Adolescents and Young Adults With Cancer: Systematic Review
Source: JMIR Cancer. 2019 Jul 31;5(2):e12071. doi: 10.2196/12071 (PMC6693302; doi:10.2196/12071)
Supplement: Multimedia Appendix 3 [file cancer_v5i2e12071_app3.pdf]

Multimedia Appendix 3

QualSyst Scores for Qualitative Papers

Author Year	QualSyst Criteria (Qualitative)										Score (%)
	Question/ Objective sufficiently described	Study design evident and appropriate	Context for the study clear	Connection to a theoretical framework/ wider body of knowledge	Sampling strategy described, relevant and justified	Data collection methods clearly described and systematic	Data analysis methods clearly described and systematic	Use of verification procedures to establish credibility	Conclusions supported by the results	Reflexivity of the account	
Ameringer et al. (2015)	2	2	2	2	2	2	2	2	2	2	100
Cantrell & Conte (2008)	1	0	2	2	0	0	0	0	1	1	35
Donovan et al. (2014)	2	2	2	2	1	2	1	0	2	0	70
Gonzalez-Morkos et al. (2014)	2	2	2	1	2	1	1	0	2	1	70
Griffiths et al. (2015)	0	1	1	1	1	1	1	1	1	1	45
Kunin-Batson et al. (2016)	2	2	2	1	1	2	1	0	2	0	65
Lai et al. (2015)	2	2	2	2	2	2	2	0	1	1	80

Author Year	QualSyst Criteria (Qualitative)										Score (%)
	Question/ Objective sufficiently described	Study design evident and appropriate	Context for the study clear	Connection to a theoretical framework/ wider body of knowledge	Sampling strategy described, relevant and justified	Data collection methods clearly described and systematic	Data analysis methods clearly described and systematic	Use of verification procedures to establish credibility	Conclusions supported by the results	Reflexivity of the account	
MacPherson et al. (2014)	2	2	2	1	2	2	1	0	2	0	70
Maurice-Stam (2014)	2	2	2	1	1	2	1	0	2	0	65
Stinson et al. (2013)	2	2	2	2	0	2	2	2	2	0	80
Stinson et al. (2015a)	2	2	2	2	0	2	2	1	2	0	75
Suzuki & Beale (2006)	2	1	2	1	1	1	1	0	1	0	50
Winterling et al. (2016)	2	2	2	2	1	1	0	0	1	1	60
Phelps et al. (2016)	2	2	2	1	1	1	0	0	2	1	55

Note. Criteria were scored either 2,1 or 0 (2=yes, 1=partial, 0=no) or if the criteria was not applicable to the paper it was scored N/A. To make them comparative, overall scores are presented as a %.
