# Supplementary material for: Digital Interventions to Support Adolescents and Young Adults With Cancer: Systematic Review
Source: JMIR Cancer. 2019 Jul 31;5(2):e12071. doi: 10.2196/12071 (PMC6693302; doi:10.2196/12071)
Supplement: Multimedia Appendix 4 [file cancer_v5i2e12071_app4.pdf]

Multimedia Appendix 4

QualSyst Scores for Quantitative Papers

Author Date		QualSyst Criteria (Quantitative)													Score (%)
	Question/ Objective sufficiently described	Study design evident and appropriate	Method of subject/ comparison group selection or source of information/ input variable described and appropriate	Subject characteristic sufficiently described	If interventional and random allocation was possible, was it described?	If interventional and blinding of investigators was possible, was it reported?	If interventional and blinding of subjects was possible, was it reported?	Outcomes and (if applicable) exposure measures well defined and robust to measurement/ misclassification bias? Means of assessment reported?	Sample size appropriate	Analytic methods described/ justified and appropriate	Some estimate of variance is reporter for the main results	Controlled for confounding	Results reported in sufficient detail	Conclusions supported by the results	
Akard et al. (2015)	2	2	2	2	2	0	N/A	2	1	2	2	0	2	2	81
Ameringer et al. (2015)	2	2	2	2	N/A	N/A	N/A	2	2	2	2	0	2	2	91
Baggott et al. (2012)	2	2	2	2	N/A	N/A	N/A	2	1	2	N/A	N/A	2	2	94
Beale et al. (2007)	2	2	2	2	0	0	N/A	2	2	2	2	1	2	2	81
Berg et al. (2014)	2	2	2	2	N/A	N/A	N/A	2	1	2	0	0	2	2	77
Blaauwbroek et al. (2012)	2	2	2	2	0	0	N/A	2	1	2	1	0	2	2	64
Burns et al. (2009)	2	2	2	2	2	0	N/A	2	1	2	1	1	2	2	73

Author Date			QualSyst Criteria (Quantitative)												Score (%)
	Question/ Objective sufficiently described	Study design evident and appropriate	Method of subject/ comparison group selection or source of information/ input variable described and appropriate	Subject characteristic sufficiently described	If interventional and random allocation was possible, was it described?	If interventional and blinding of investigators was possible, was it reported?	If interventional and blinding of subjects was possible, was it reported?	Outcomes and (if applicable) exposure measures well defined and robust to measurement/ misclassification bias? Means of assessment reported?	Sample size appropriate	Analytic methods described/ justified and appropriate	Some estimate of variance is reporter for the main results	Controlled for confounding	Results reported in sufficient detail	Conclusions supported by the results	
Emmons et al. (2013)	2	2	2	2	2	0	N/A	2	2	2	2	2	2	2	92
Gilliam et al. (2011)	2	2	2	2	N/A	N/A	N/A	2	0	2	0	0	2	2	73
Hardy et al. (2011)	2	1	1	1	N/A	N/A	N/A	2	0	2	0	0	1	1	50
Hooke et al. (2016)	2	2	2	2	0	0	N/A	2	1	2	0	0	2	2	68
Huang et al. (2014)	2	2	2	2	2	N/A	N/A	2	1	1	0	1	2	2	79
Jibb et al. (2017)	2	2	2	2	N/A	N/A	N/A	2	2	2	0	0	2	2	82
Jones et al. (2010)	2	2	2	2	1	0	N/A	2	1	2	0	0	2	2	69
Kato et al. (2008)	2	2	2	2	2	2	N/A	2	2	2	2	2	2	2	100

Multimedia Appendix 4

Author Date		QualSyst Criteria (Quantitative)													Score (%)
	Question/ Objective sufficiently described	Study design evident and appropriate	Method of subject/ comparison group selection or source of information/ input variable described and appropriate	Subject characteristic sufficiently described	If interventional and random allocation was possible, was it described?	If interventional and blinding of investigators was possible, was it reported?	If interventional and blinding of subjects was possible, was it reported?	Outcomes and (if applicable) exposure measures well defined and robust to measurement/ misclassification bias? Means of assessment reported?	Sample size appropriate	Analytic methods described/ justified and appropriate	Some estimate of variance is reporter for the main results	Controlled for confounding	Results reported in sufficient detail	Conclusions supported by the results	
Kesler et al. (2011)	1	1	2	2	N/A	N/A	N/A	2	0	2	1	2	2	2	77
Knijnenburg et al. (2012)	2	1	N/A	2	N/A	N/A	N/A	1	1	2	1	0	1	1	60
McLaughlin et al. (2012)	2	2	2	2	N/A	N/A	N/A	2	1	2	0	N/A	2	2	85
Mendoza (2017)	1	2	2	2	0	0	N/A	2	2	2	0	0	2	1	62
Rabin et al. (2011)	2	2	1	1	0	N/A	N/A	1	1	2	0	0	1	2	46
Rodgers et al. (2014)	2	2	2	1	N/A	N/A	N/A	1	0	2	0	0	1	1	43
Sabel et al. (2016)	2	2	2	2	2	2	N/A	2	1	2	2	2	2	2	96
Seitz et al. (2014a)	2	2	2	2	1	0	N/A	2	1	2	2	2	2	2	85

Author Date			QualSyst Criteria (Quantitative)												Score (%)
	Question/ Objective sufficiently described	Study design evident and appropriate	Method of subject/ comparison group selection or source of information/ input variable described and appropriate	Subject characteristic sufficiently described	If interventional and random allocation was possible, was it described?	If interventional and blinding of investigators was possible, was it reported?	If interventional and blinding of subjects was possible, was it reported?	Outcomes and (if applicable) exposure measures well defined and robust to measurement/ misclassification bias? Means of assessment reported?	Sample size appropriate	Analytic methods described/ justified and appropriate	Some estimate of variance is reporter for the main results	Controlled for confounding	Results reported in sufficient detail	Conclusions supported by the results	
Seitz et al. (2014b)	2	2	2	2	1	0	N/A	2	1	2	2	2	2	2	85
Stinson et al. (2015b)	2	2	2	2	2	2	N/A	2	2	2	2	2	2	2	100
Valle et al. (2013)	2	2	2	2	2	N/A	N/A	2	1	2	0	0	2	2	68
Valle et al. (2015a)	2	2	2	1	1	N/A	N/A	1	1	2	0	0	2	2	58
Wiklander et al. (2017)	2	2	N/A	2	N/A	N/A	N/A	2	1	2	1	0	2	2	80
Wint et al. (2002)	2	2	1	2	0	N/A	N/A	1	2	2	2	0	1	2	71
Wu et al. (2011)	2	2	1	2	N/A	N/A	N/A	2	2	2	0	N/A	1	2	73

Note. Criteria were scored either 2,1 or 0 (2=yes, 1=partial, 0=no) or if the criteria was not applicable to the paper it was scored N/A. To make them comparative, overall scores are presented as a %.
