# Supplementary material for: Digital Interventions to Support Adolescents and Young Adults With Cancer: Systematic Review
Source: JMIR Cancer. 2019 Jul 31;5(2):e12071. doi: 10.2196/12071 (PMC6693302; doi:10.2196/12071)
Supplement: Multimedia Appendix 5 [file cancer_v5i2e12071_app5.pdf]

Multimedia Appendix 5

Mode of Delivery

	Automated Functions			Communicative Functions			Supplementary Modes of Communication					
	Instinctive functions embedded within the programme			Functions which provide the opportunity for connection or communication with another person			Additional modes of delivering the intervention content					
**Author, Year of Publication**<br>**Intervention Name**	a) The use of an enriched information environment	b) Automated tailored feedback based on individual progress monitoring	c) Automated follow up messages	a) Access to an advisor to request advice (e.g. 'ask the expert facility', expert led discussion, or chat session.	b) Scheduled contact with advisor (e.g. appointment or scheduled emails)	c) peer-to-peer access (e.g. buddy systems, peer-to-peer discussion boards)	a) email	b) telephone	c) short messaging service (SMS)	d) CD-ROM	e) videoconferencing	f) Other
* No identifiable mode of delivery as per these criteria identifiable from text description in paper												
**Websites**												
Seitz et al., 2014a; Seitz et al., 2014b<br><br>Onco-STEP												
Phelps et al., 2016<br><br>C:EVOLVE												
Lai et al., 2015<br><br>SyMon-SAYS												
Kunin-Batson et al., 2016												
Knijnenberg et al., 2013*												
Hardy et al., 2011<br><br>Captain's Log												
Berg et al., 2014<br><br>No study name												
Blaauwbroek et al., 2011<br><br>No study name												
Cantrell & Conte 2009<br><br>Hope Intervention Programme (HIP)												

Multimedia Appendix 5

	**Automated Functions**			**Communicative Functions**			**Supplementary Modes of Communication**					
	**Instinctive functions embedded within the programme**			**Functions which provide the opportunity for connection or communication with another person**			**Additional modes of delivering the intervention content**					
**Author, Year of Publication** **Intervention Name**	a) The use of an enriched information environment	b) Automated tailored feedback based on individual progress monitoring	c) Automated follow up messages	a) Access to an advisor to request advice (e.g. 'ask the expert facility', expert led discussion, or chat session.	b) Scheduled contact with advisor (e.g. appointment or scheduled emails)	c) peer-to-peer access (e.g. buddy systems, peer-to-peer discussion boards)	a) email	b) telephone	c) short messaging service (SMS)	d) CD-ROM	e) videoconferencing	f) Other
* No identifiable mode of delivery as per these criteria identifiable from text description in paper												
Emmons et al., 2013<br><br>Partnership for Health 2 (PFH-2)	■	■		■			■					
Gonzalez-Morkos et al., 2014<br><br>TI webcast						■		■			■	
Griffiths et al., 2015<br><br>Realshare	■			■		■	■	■				
Huang et al., 2014<br><br>Fit4Life	■		■	■	■			■	■			
McLaughlin et al., 2011<br><br>LIFE Community	■		■			■			■			
Rabin et al., 2011<br><br>Step into Motion (SIM) website	■	■	■	■		■	■					
Suzuki & Beale 2006<br><br>No study name										■		■
Valle et al., 2013; Valle et al., 2015<br><br>FITNET	■	■	■	■	■	■						
Wiklander et al., 2017<br><br>Fex-Can	■		■	■	■		■					

Multimedia Appendix 5

	Automated Functions			Communicative Functions			Supplementary Modes of Communication					
	Instinctive functions embedded within the programme			Functions which provide the opportunity for connection or communication with another person			Additional modes of delivering the intervention content					
**Author, Year of Publication**<br>**Intervention Name**	a) The use of an enriched information environment	b) Automated tailored feedback based on individual progress monitoring	c) Automated follow up messages	a) Access to an advisor to request advice (e.g. 'ask the expert facility', expert led discussion, or chat session.	b) Scheduled contact with advisor (e.g. appointment or scheduled emails)	c) peer-to-peer access (e.g. buddy systems, peer-to-peer discussion boards)	a) email	b) telephone	c) short messaging service (SMS)	d) CD-ROM	e) videoconferencing	f) Other
* No identifiable mode of delivery as per these criteria identifiable from text description in paper												
Winterling et al., 2016<br><br>Fex-Can												
Wu et al., 2012*<br><br>Electronic Self-report Assessment Cancer (ESRA-C)												
**Mobile Applications**												
Baggott et al., 2012<br><br>mOST												
Macpherson et al., 2014; Ameringer et al., 2015<br><br>C-SCAT												
Rodgers et al., 2013 & Rodgers et al., 2014<br><br>EAT!												
Jibb et al., 2017<br><br>PainSquad+												
Stinson et al., 2013; Stinson et al., 2015a; Stinson et al., 2015b<br><br>Pain Squad												
**Other (Social Media, PC game, video, CD-ROM, Wearable, Virtual Reality, Computer Program, e-Mail)**												

Multimedia Appendix 5

	Automated Functions			Communicative Functions			Supplementary Modes of Communication					
	Instinctive functions embedded within the programme			Functions which provide the opportunity for connection or communication with another person			Additional modes of delivering the intervention content					
**Author, Year of Publication**<br>**Intervention Name**	a) The use of an enriched information environment	b) Automated tailored feedback based on individual progress monitoring	c) Automated follow up messages	a) Access to an advisor to request advice (e.g. 'ask the expert facility', expert led discussion, or chat session.	b) Scheduled contact with advisor (e.g. appointment or scheduled emails)	c) peer-to-peer access (e.g. buddy systems, peer-to-peer discussion boards)	a) email	b) telephone	c) short messaging service (SMS)	d) CD-ROM	e) videoconferencing	f) Other
* No identifiable mode of delivery as per these criteria identifiable from text description in paper												
Valle et al., 2013; Valle et al., 2015<br>FITNET				■							■	
Mendoza, 2017		■			■	■			■			
Kesler et al., 2011							■					
Jones et al., 2010<br>Conquering Cancer Network	■	■								■		
Hooke et al., 2016<br>Fitbit			■	■				■	■			
Gilliam et al., 2011					■	■						
Donovan et al., 2014						■						
Akard et al., 2015*												
Beale et al., 2007; Kato et al., 2008<br>Re-Mission												■
Burns et al., 2009*<br>TMV												

Multimedia Appendix 5

	**Automated Functions**			**Communicative Functions**			**Supplementary Modes of Communication**					
	**Instinctive functions embedded within the programme**			**Functions which provide the opportunity for connection or communication with another person**			**Additional modes of delivering the intervention content**					
**Author, Year of Publication** **Intervention Name**	a) The use of an enriched information environment	b) Automated tailored feedback based on individual progress monitoring	c) Automated follow up messages	a) Access to an advisor to request advice (e.g. 'ask the expert facility', expert led discussion, or chat session.	b) Scheduled contact with advisor (e.g. appointment or scheduled emails)	c) peer-to-peer access (e.g. buddy systems, peer-to-peer discussion boards)	a) email	b) telephone	c) short messaging service (SMS)	d) CD-ROM	e) videoconferencing	f) Other
* No identifiable mode of delivery as per these criteria identifiable from text description in paper												
Sander Wint et al., 2002* Virtual reality glasses												
